# Review of "Pediatric Hydrocephalus" by Guiseppe Cinalli, Wirginia Maixner, Christian Sainte-Rose (editors)

**DOI:** 10.1186/1743-8454-3-3

**Published:** 2006-03-22

**Authors:** Hazel C Jones

**Affiliations:** 1 Department of Pharmacology, University of Florida, Gainesville, FL 32610, USA; 2 Gagle Brook House, Chesterton, Bicester, Oxon OX26 1UF, UK

## Abstract

This review summarizes the content and usefulness of this multi-author volume for those involved in the treatment of pediatric hydrocephalus.

## 

Disorders of the central nervous system can be very distressing at any time of life, but particularly so when they affect children. Hydrocephalus is the most common problem encountered by pediatric neurosurgeons and is a disorder for which there is no absolute cure. Hydrocephalus can be broadly defined as enlargement of the cerebral ventricles with an excess of cerebrospinal fluid (CSF). It has many different causes but the most usual are obstructions of various types in the CSF flow pathway.

Progress in basic biomedical research has provided new and compelling evidence for a fundamental role of the CSF in promoting and maintaining brain function. It is important during development where it provides vital growth factors and signals for the control of neurogenesis [1]. It has essential roles in the mature brain for homeostatic control of the brain fluid environment [2], for neuroendocrine signaling [3] and for migration of neuroblasts to the olfactory bulb [4]. CSF function is also very important in the aging brain, which is prone to neurological disease and age-related dementia [5,6]. It follows, therefore, that when normal CSF flow and chemical composition are disturbed in hydrocephalus, there are adverse effects in the brain. The subtleties of the consequences of abnormal CSF flow and composition are only beginning to be understood and there is no doubt that much more will be revealed in the future. These facts make it imperative that patients with hydrocephalus are diagnosed and treated in a timely and appropriate manner. Because of the nature of the condition, treatment for hydrocephalus is necessarily directed towards alleviation of the signs and symptoms by neurosurgical methods. Optimal outcome also requires appropriate patient management throughout life.

'Pediatric Hydrocephalus' is a specialist book written by clinicians for clinicians. It is directed at neurosurgeons, pediatric neurologists and pediatricians and will also be of interest to radiologists, pathologists and clinical geneticists. It draws upon the clinical experience of fifty-eight experts. The subject matter is divided into thirty-three chapters and covers the following aspects of hydrocephalus: genetics, CSF physiology, pathophysiology, classification, and imaging techniques together with twelve chapters on the different types of hydrocephalus, four chapters on shunt hardware, surgery and complications, and eight chapters on endoscopy and third ventriculostomy. Long-term outcome and behavioral aspects of hydrocephalic children are not presented in any detail.

Shunt treatment for hydrocephalus was introduced into practice in the 1960s, when it significantly reduced the mortality rate. Since then, there have been numerous developments with improved diagnosis and innovations in neurosurgical techniques. This book gives a modern perspective on all aspects of treatment. The chapters on types of hydrocephalus with different etiologies include the diagnosis, treatment and outcomes for posthemorrhagic hydrocephalus, hydrocephalus due to tumors and other space-occupying lesions, hydrocephalus associated with myelomeningocele and with Dandy-Walker malformation, with parasitic and fungal disease and with aqueductal stenosis. The principles of shunt design and insertion techniques are explained, as are complications such as slit ventricle syndrome, shunt infections and shunt malfunction, together with abdominal problems at the distal end. It is good to see a discussion on the relative merits of shunt versus endoscopy treatments, thus bringing the newer endoscopic techniques into perspective. The advantages of modern imaging techniques both for diagnosis and post-operative assessment are emphasized. The two final chapters cover the specific complications of endocrine disturbances and epilepsy in hydrocephalic patients. Throughout the book, the text is clearly presented with subheadings and high-quality illustrations.

In summary, this book will be of great benefit to both aspiring and practicing neurosurgeons worldwide.

## Competing interests

The author(s) declare that they have no competing interests.

## Authors' contributions

Sole author.
